# Smartphone users: Understanding how security mechanisms are perceived and new persuasive methods

**DOI:** 10.1371/journal.pone.0173284

**Published:** 2017-03-15

**Authors:** Mansour Alsaleh, Noura Alomar, Abdulrahman Alarifi

**Affiliations:** 1 King Abdulaziz City for Science and Technology (KACST), Riyadh, Kingdom of Saudi Arabia; 2 Software Engineering Department, King Saud University, Riyadh, Kingdom of Saudi Arabia; University of Texas at San Antonio, UNITED STATES

## Abstract

Protecting smartphones against security threats is a multidimensional problem involving human and technological factors. This study investigates how smartphone users’ security- and privacy-related decisions are influenced by their attitudes, perceptions, and understanding of various security threats. In this work, we seek to provide quantified insights into smartphone users’ behavior toward multiple key security features including locking mechanisms, application repositories, mobile instant messaging, and smartphone location services. To the best of our knowledge, this is the first study that reveals often unforeseen correlations and dependencies between various privacy- and security-related behaviors. Our work also provides evidence that making correct security decisions might not necessarily correlate with individuals’ awareness of the consequences of security threats. By comparing participants’ behavior and their motives for adopting or ignoring certain security practices, we suggest implementing additional persuasive approaches that focus on addressing social and technological aspects of the problem. On the basis of our findings and the results presented in the literature, we identify the factors that might influence smartphone users’ security behaviors. We then use our understanding of what might drive and influence significant behavioral changes to propose several platform design modifications that we believe could improve the security levels of smartphones.

## 1 Introduction

The mobility, portability, and increasing capabilities of smartphones have significantly contributed to the increasing popularity of these multi-purpose devices. A recently published report showed that more than two thirds of American adults possess a smartphone [1]. Furthermore, more than 334 million smartphones were sold in less than 3 months in 2015 [2]. As smartphones begin to replace personal computers because of their advanced features and ease of use, large volumes of sensitive data are now stored and processed in smartphones including contacts, emails, photos, and videos. This makes smartphones an attractive target for hackers, particularly as regards the many ways of installing malicious codes on their victims’ devices and gaining unauthorized access to users’ sensitive data [3–5]. For example, the lack of awareness of many smartphone users might make them vulnerable to downloading malicious applications from uncontrolled app marketplaces [4, 6]. Receiving phishing messages that could come from SMS, MMS, email messages, social networks, and phone calls might also increase the vulnerability of smartphone users to many security- or privacy-related threats [4].

In 2014, Symantec analyzed over 6 million mobile applications and found that over 15% of them included malicious content, whereas around 37% were considered grayware programs [5]. Recently, Apple also stated that 100 billion applications were downloaded from its application repository [7]. A report published by Symantec also showed that smartphone vulnerabilities experienced a growth of 32% in 1 year [5].

A recent survey [8] showed that there is a lack of awareness among smartphone users about the security and privacy risks associated with downloading smartphone apps. Most surveyed participants assumed that controlled app marketplaces (e.g., Google Play) are secure [8], which indicates that users’ perceptions might negatively affect their security. According to the results of another survey [5], most smartphone users have worries and fears related to their privacy and security, yet they perform risky behaviors (e.g., over 65% of the surveyed individuals gave free applications permissions to access their data). Understanding smartphone users’ perceptions and misconceptions about security and privacy is therefore essential. This will help researchers develop mechanisms that preserve the confidentiality, integrity, and availability of data stored in smartphones.

In this paper, thirty qualitative interviews were performed to understand users’ security-related behaviors and to examine the correlations between them. We extend the work of Egelman et al. [9] by identifying the correlations and dependencies between various privacy- and security-related behaviors (in addition to locking behaviors), and by understanding how they are influenced by users’ preconceived perceptions, and propose new persuasive approaches. One of our interesting findings shows that the behavior related to locking mechanisms correlates with the practices related to backing up smartphone data, saving photos in gallery applications and connecting to public Wi-Fi hotspots. For example, we found that over 88% of our participants who chose not to lock their phones do not back up their smartphone devices. Furthermore, at least eight out of nine users who do not use smartphone locking mechanisms connect to public Wi-Fi networks. Similarly, more than 88% of the users who do not lock their phones save their personal photos in gallery applications. Inspired by the participants’ subjective feedback related to many security features as well as by the related behavioral literature, we identify the factors that could assist in predicting smartphone users’ decisions of whether to adopt protective behaviors or not.

**Contributions.** Our main contributions are the following.

1. **Studying Users’ Behaviors Toward Multiple Security Features.** Our study examined smartphone users’ behaviors toward seven different security features including locking mechanisms, application repositories, smartphone location services, and mobile instant messaging. To the best of our knowledge, ours is the first study to explore such questions and the first to provide an insight into the correlations between different security- and privacy-related behaviors.
2. **Establishing a Security Model.** On the basis of our findings and the results of past studies, we present a model that demonstrates the factors that may negatively or positively shape and influence the self-protective behaviors of smartphone users.
3. **Platform Design Recommendations.** By considering the factors that might impact smartphone users’ adoption of more secure behaviors, we discuss possible adjustments and design modifications that could be applied to improve the security of smartphone platforms.

**Organization.** We begin by formulating the research hypotheses in Section 2. The study methodology followed in our structured interviews is then described in Section 3. This is followed by explaining our participants’ security behaviors and summarizing their reasons behind the adoption of the observed behaviors in Sections 4 and 5. The results and analyses of the conducted interviews are presented in Section 6. Section 7 discusses several smartphone platform design modifications. We then present a synthesis of related past studies in Section 8. Section 9 offers directions for future research and describes the limitations of our study. Section 10 concludes the paper.

## 2 Theoretical foundation and hypotheses

Prior behavioral research has emphasized the importance of studying users’ motivations toward adopting protective technologies (e.g., anti-viruses and firewalls) and analyzing the factors that drive users to protect themselves [10]. Studying the behavioral factors that affect individuals’ compliance to security policies in organizational settings has also been given significant focus in the literature [11, 12]. Balebako et al. considered the problem from the perspective of application developers by examining the barriers that prevent the engagement of developers in security and privacy best practices [13]. In the psychology literature, there are a number of behavioral theories that are widely used to explain the behavioral patterns of individuals and the factors that would positively or negatively affect their decisions to take protective or preventative actions. To understand the motives behind the voluntary involvements of smartphone users in self-protective behavior, we formulated a set of hypotheses based on the theoretical foundations discussed in the literature.

As we seek to obtain a complete understanding of smartphone users’ perceptions and attitudes toward avoiding a number of IT threats, we chose to base the theoretical foundation of this study on the principles of the Technology Threat Avoidance Theory (TTAT). TTAT synthesizes the benefits of a number of well-established behavioral models and theories including the Protection Motivation Theory (PMT), which has been widely applied to understand how people perceive and cope with security- and privacy-related threats [14–21]. It is also worth noting that TTAT was specifically designed to determine the factors that lead individuals to avoid IT threats by taking protective actions [15]. According to TTAT, users’ decisions to avoid IT threats are mainly linked to their perceptions of the severity and vulnerability of these threats, their subjective assessments of the effectiveness and the costs of available safeguarding measures, and their confidence in their abilities to adopt these measures for taking protective actions [15, 22]. Based on the principles of TTAT and PMT, users’ decisions to undertake protective actions against IT threats result from their subjective assessments of how harmful those threats are and their likelihood of experiencing them [18]. Consequently, the more the users feel that they are at risk or that the chances of being affected by a given IT threat are high, the more likely they will be motivated to engage in protective actions [22].

The consideration of perceived threat severity and susceptibility has been applied to predict users’ intentions to adopt safe computing behaviors in different contexts [17, 18, 20]. For instance, Tu et al. suggested that the more individuals consider themselves threatened by the negative consequences of losing their mobile devices, the better chance there is that they will protect themselves against mobile theft or loss [17]. Matt et al. also found that users’ adoption of privacy-enhancing technologies can be predicted from their perceptions of the degree of harm that they would face as a result of a privacy-invading incident [20]. To the best of our knowledge, existing literature has not sufficiently investigated whether a relationship exists between smartphone users’ perceptions of threat and their attitudes toward avoiding privacy and security threats. Therefore, we propose that:

**H1:** The perceived threat of security or privacy incidents can affect the actual behavior of smartphone users.

According to TTAT, individuals also weigh the costs and benefits associated with taking protective actions before deciding to change their behavior [15]. Perceptions of time, financial cost, and cognitive effort of adopting a safe computing behavior are all important predictors of users’ decisions to protect themselves [14, 15, 18]. Therefore, we hypothesize that:

**H2:** Smartphone users are more likely to engage in security or privacy protective behavior as the costs associated with adopting such behavior decrease.

It has also been shown that technology awareness affects users’ decisions of whether to adopt protective technologies or not [14, 22, 23]. This includes users’ awareness of IT threats, the negative consequences of these threats, and possible countermeasures that can be used to combat such threats [14, 23]. Dinev et al. proposed to extend the Theory of Planned Behavior (TPB) [24], which has been widely applied to explain human behavior, to include technology awareness as a main predictor of individuals’ behavioral intentions [23]. After applying PMT to understand the security behaviors of computer users, Hanus et al. also found that technology awareness is an important predictor of users’ self-protective behaviors [14]. TTAT also suggests that individuals’ security awareness directly impacts their perceptions of IT threats [15]. To explore the role of technology awareness in shaping smartphone users’ self-protective behaviors, we propose that:

**H3:** Smartphone users’ awareness of technology positively impacts their security and privacy behavior.

Although a great deal of research has focused on understanding the motives behind users’ intentions to behave safely and avoid threats of negative technologies such as viruses, we note that the literature lacks studies that determine whether there are correlations between a user’s different security- or privacy-related behaviors. We seek to understand whether users’ decisions to protect themselves from certain security or privacy threats may lead them to protect themselves from other threats. Locking smartphones, being careful when connecting to public Wi-Fi networks, and making backup copies of stored data are only a very few examples of behaviors adopted by smartphone users. As smartphones are nowadays being used for a variety of purposes, users might also choose to behave securely in some situations and accept the risks associated with adopting other less protective behaviors. We expect studying the possible links between behaviors to be the first step toward understanding the properties of different user habits and the conditions under which smartphone users choose to protect themselves. This would ultimately lead to simplifying the design of protective technologies that have better chances of being accepted by smartphone users. Therefore, we believe that users’ different threat avoidance habits correlate with each other.

## 3 Methodology

This section describes the methodology we followed for testing the hypotheses defined in Section 2. Thirty structured qualitative interviews were conducted with users for understanding their risk perceptions and attitudes toward adopting smartphone-related security features. The responses were analyzed qualitatively and quantitatively to understand the reasons behind making decisions that might lead individuals to behave in a less protective manner.

### 3.1 Participant recruitment

A quota sampling approach was used to recruit participants for this study in which the selection was based on predefined criteria. The reason for following this recruitment approach was to obtain a sample that represented smartphone users with varying backgrounds, educational levels, and demographics. For this reason, our sample had different age groups ranging from 18 to 48 years. Furthermore, diversity in occupation, gender, marital status, and income level was assured. The selected sample only included users who had been using smartphones for more than 6 months and excluded participants whose ages were under 16 years. The conducted user study has been reviewed and approved by KACST Experimentation Ethics Committee. To keep identities of participants anonymous, verbal informed consent to participate in this study has been obtained from each participant. Prior to the start of each interview session, a voluntary agreement to participate in this research study was given to each participant to verbally read the elements and verbally agrees to participate. The agreement includes purpose of the study, procedures, potential discomforts, potential benefits of the research, confidentiality in protecting participants’ information, and rights of research subjects. We labeled them anonymously and marked those who agreed to participate. The ethics committee approved the consent procedure. More details about the recruited participants are shown in Table 1.

**Table 1:** Demographics of the interviewed participants.

Parameter	Results
Age	Mean: 30 years
	Standard deviation: 10
	Minimum: 18 years
	Maximum: 48 years
Gender	Male: 43%
	Female: 57%
Income level	Mean: 3655 USD/year
	Standard deviation: 1487
	Minimum: 1867 USD/year
	Maximum: 8000 USD/year
Occupation	Student: 23%
	Unemployed: 17%
	Doctor: 13%
	Teacher: 13%
	HR Employee: 10%
	Administrative: 7%
	Professor: 3%
	Accountant: 3%
	Business man: 3%
	other: 3%
Residential type	Urban: 80%
	Suburban: 7%
	Rural: 13%

### 3.2 Procedure overview

**Fig 1:**
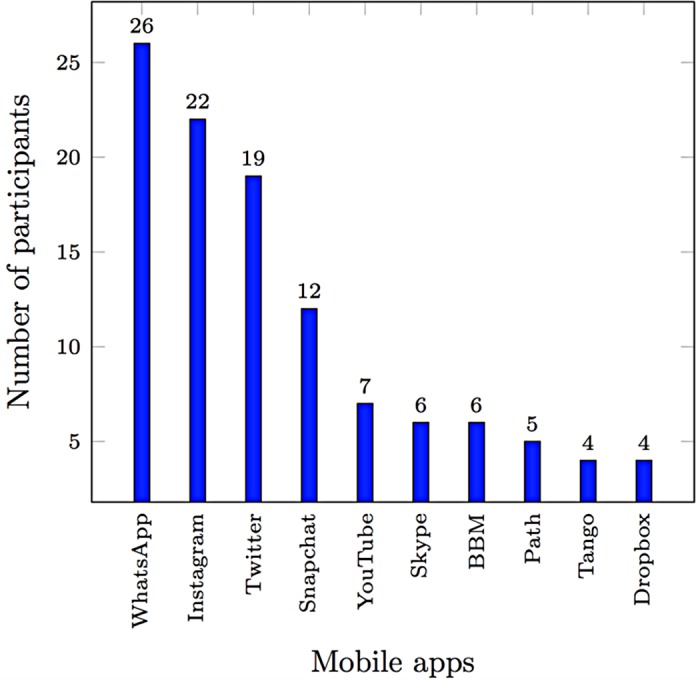
Participants’ most commonly used smartphone apps.

Thirty qualitative interviews were conducted individually to examine the risk perceptions of smartphone users. Interview duration varied from an hour to an hour and a half. The questionnaire we used in our interviews consisted of nine categories that were set to examine participants’ security risk perceptions related to the sensitivity of stored data and their overall smartphone usage. The questionnaire included questions that were clear, simple, understandable, and consistent. We gathered responses related to locking, sharing, synchronization, and backup behaviors. In general, our aim was to understand how various risk perceptions affected users’ security behavior and the link between different security- and privacy-related behaviors. In addition to the perceptions examined in the literature (e.g., [9]), we investigated the security behaviors related to the following additional areas (see [25] for more details about the interview questions):

1. **Mobile Messaging: WhatsApp Messenger.** As more than 87% of our participants indicated that they use WhatsApp Messenger, one of the most popular instant messaging platforms on smartphones, we included WhatsApp Messenger in our study to gain a better understanding of our participants’ approaches to chatting (see Fig 1). For example, we asked the interviewed participants whether they share their photos or locations with strangers. They were also asked whether they chat with people who are not listed in their contact lists, whether they trust the messages that they receive, and whether they use a specific mechanism for validating the security of received content (such as clicking on received hyperlinks that may contain phishing or malware content). Their level of awareness was also examined by asking whether they think that the messages sent through WhatsApp are encrypted and whether WhatsApp records their conversations.
2. **Smartphone Application Repositories.** This category investigated the risk perceptions and security behaviors related to downloading applications from smartphone application markets. For example, we first asked participants whether smartphone applications’ permission requests affect their installation decisions; second, whether they use specific malware or spyware protection and prevention programs; and, third, whether they install apps from uncontrolled app marketplaces.
3. **Gallery and Camera Applications.** To understand the interviewees’ perceptions and behaviors related to storing and sharing their personal photos, we asked whether they upload their photos to cloud-based storage systems (such as Dropbox and iCloud). They were also asked whether they trust particular camera applications and whether they allow social networking applications to access their photo gallery applications immediately (i.e., granting gallery access permission during installation without requiring explicit user permission for each request). Their reactions toward losing photos stored in their smartphones were investigated too.
4. **Public Wi-Fi Networks.** Participants were asked whether they connect to Wi-Fi networks in public places. We also examined their level of awareness of the risks associated with disclosing the passwords of their networks to other people and accessing sensitive information while they are connected to public Wi-Fi networks.
5. **Location Services.** The interviewed smartphone users were asked whether they use services that identify their locations while they are connected to wireless networks. They were also asked whether they use such services to locate other people. Furthermore, we asked them whether they share their location information in social networking applications and whether they know the privacy consequences of such practice.

## 4 Smartphone users: Adopted security behaviors

**Fig 2:**
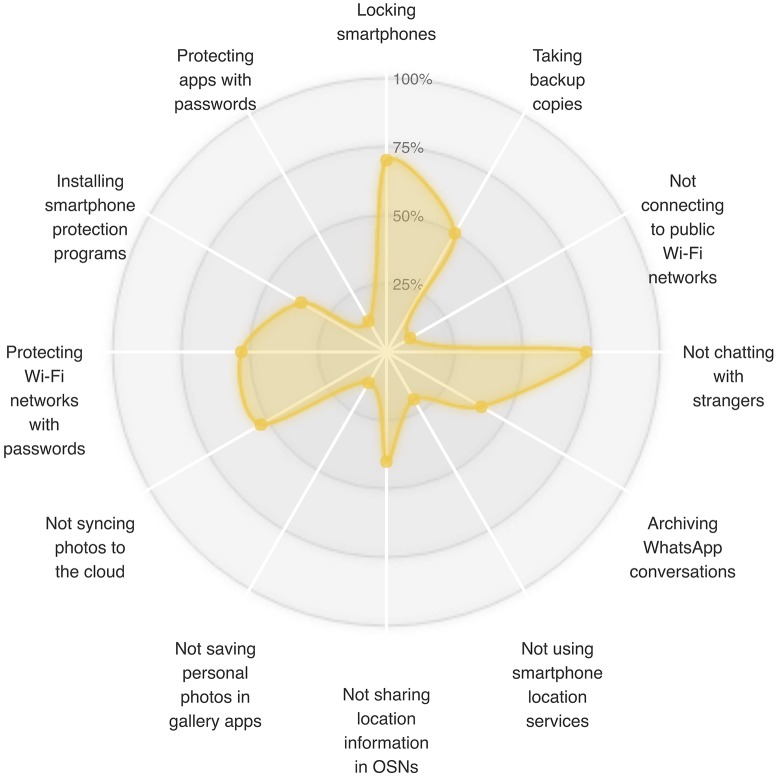
Percentages of participants who adopted each examined self-protective behavior.

In our interviews, we examined participants’ behaviors related to the use of many smartphone features. This section presents the results of our analysis and discusses our participants’ attitudes toward a set of security- and privacy-related threats. Table 2 and Fig 2 show the distributions of participants who adopted the examined security- and privacy-related behaviors.

**Table 2:** Number of participants who adopted the examined security behaviors.

Behavior	N = 30
Smartphone locking behavior	21
Smartphone backup behavior	15
Chatting with unknown people	8
Archiving WhatsApp conversations	12
Using WhatsApp location services	24
Sharing location information in online social networking sites	18
Saving personal photos in gallery apps	26
Syncing photos to the cloud	14
Protecting Wi-Fi networks with passwords	16
Connecting to public Wi-Fi networks	27
Installing smartphone protection programs	11
Protecting mobile apps with passwords	4

### 4.1 Smartphone locking

**Fig 3:**
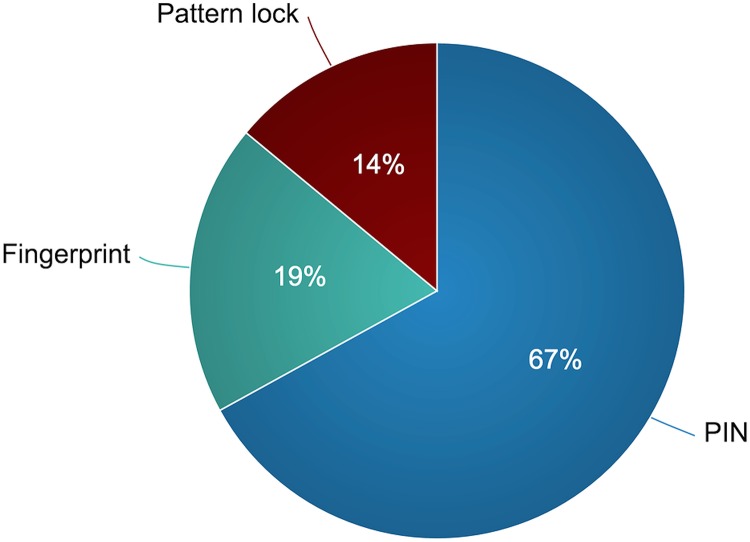
The locking mechanisms adopted by the participants.

Our observations showed that 70% of the interviewed smartphone users lock their phones. When they were asked about locking mechanisms that they employ, 14 participants indicated that they used numerical PINs, 4 participants used fingerprint authentication, and 3 participants used pattern locks (see Fig 3). Of those who chose PIN authentication, nine participants indicated that they link their passcodes to important dates or phone numbers. We also observed that the default screen timeout intervals were modified by 11 participants compared to those who locked their phones. Nine participants decreased them to 15 seconds, whereas two participants made them longer.

Interestingly, 43% of the interviewed participants indicated that they knew the security locks (e.g., PINs) of other people including their friends or family members. Furthermore, 47% of the participants who locked their phones stated that they did so to prevent strangers from accessing their phones, whereas only 19% did not want to allow their family members specifically to access their phones. Additionally, 36% of the participants utilized locking mechanisms because they did not want their children to use their smartphones (e.g., because they might make unintended phone calls).

### 4.2 Smartphone backups

**Fig 4:**
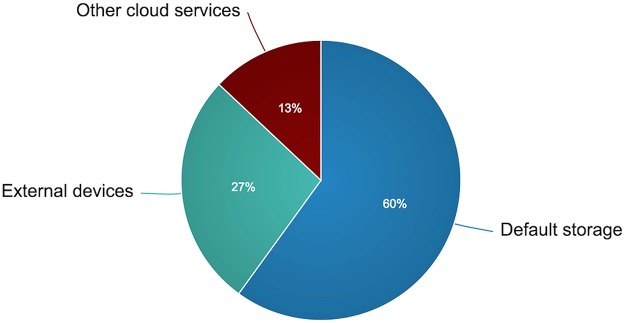
More details on the participants’ backup behavior.

By examining the backup behavior of our participants, our survey found that half of our participants did not use backup copies from their previous smartphones when they purchased their new devices except for data stored in their online accounts (e.g., Google data migration service) and that they did not transfer data from their old phones to the new ones. Four participants indicated that they saved backup copies of their smartphone data in their personal computers or their tablet devices, whereas only 13% took advantage of the currently available cloud-based storage systems such as Dropbox and Google Drive (see Fig 4). Further, the majority of the interviewees who backed up their data (9 out of 15) use the default cloud storage services provided by their mobile platforms (e.g., iCloud for iPhone devices). When asked about their reasons for not utilizing the advantages of these systems, 67% of our participants indicated that they had never heard about these systems. They were also asked whether they are willing to pay for a service that backs up their data regularly, and only three participants thought it was worth paying for. We also observed that four participants did not realize the importance of backing up their data before experiencing data loss incidents, whereas 11 participants had begun to back up their data since buying new smartphones.

### 4.3 Mobile messaging: WhatsApp

To understand the security and privacy threats related to WhatsApp chatting, we gathered data about our participants’ willingness to chat with people who are not listed in their contact lists. Our results showed that 73% of our participants indicated that they do not chat with unknown people. We also found that 60% of the participants do not archive their WhatsApp conversations because they believe that the content exchanged in their conversations is not important. Furthermore, only 23% of our participants trust the content received via WhatsApp. To examine the awareness of our participants, we asked whether they thought that data transmitted via WhatsApp is encrypted or not. Surprisingly, 33% of them did not know, whereas 57% thought that WhatsApp does not employ data encryption mechanisms.

### 4.4 Smartphone location services

Although 80% of the interviewed participants use smartphone location services, the majority indicated that they do not always turn on these services. The results also showed that about half of the participants use smartphone location services to locate other people. By examining the location-sharing behavior of our participants, we found that 60% do share their locations on social networking sites. Furthermore, we found that 57% of them felt that sharing their location information with others does not bring in any security- or privacy-related risks. In the WhatsApp messaging platform, 38% stated that they use the WhatsApp location service to share their locations with everyone. Furthermore, 30% indicated that they share their location with people they know, whereas only 20% of the participants stated that they do not use the said service.

### 4.5 Smartphone application stores

To understand the participants’ behavior toward using the smartphone application markets, we examined their interests in downloading protection applications that alert them whenever they download anything harmful. The results showed that 67% of our participants were interested, whereas the remainder either thought that these programs are unnecessary or did not understand their purpose. Despite that, the smartphones owned by 19 out of the 30 participants had no protection programs. When considering data access permission requests, 53% of the participants indicated that they do not pay attention to these requests when installing applications. Interestingly, some participants were unaware of the risks that might come as a result of granting permissions to smartphone apps. Furthermore, 19 participants indicated that their installation decisions are affected by applications’ permission requests. Fig 1 summarizes our participants’ most commonly used smartphone applications.

### 4.6 Gallery and camera applications

Eighty-seven percent of the interviewed smartphone users store personal photos in the default gallery and camera smartphone applications. The others who do not save their personal photos in their smartphones said that they save them in external hard disks or flash memory cards. Around 40% of our participants stated that they save their highly sensitive photos for at most a month, whereas the remaining users did not consider their saved photos sensitive. Furthermore, 53% of the participants indicated that they do not sync their photos to the cloud. We also found that 80% of the users interviewed do not use other gallery programs because 43% of them believe that these programs are not secure. When asked about the feature that allows a smartphone user to snap a picture without opening the device, 47% stated their discomfort because they do not want other people to use their smartphones without their permissions.

### 4.7 Wi-Fi networks

Our observations show that 43% of the participants indicated that their Wi-Fi passwords are known to other people such as their friends and colleagues. We also observed that the common Wi-Fi networks used by 47% of the interviewees were not protected with passwords and could be accessed by anyone. Surprisingly, 90% of the participants connect to public Wi-Fi networks. Furthermore, 40% of our participants were concerned about their privacy while they are connected to public wireless networks, whereas 47% were either not particularly concerned or had never thought about it.

### 4.8 Passwords

During our interviews, we observed that the mobile applications used by 87% of our participants can be accessed anytime without the need for passwords. Furthermore, 21 participants indicated that they entered the passwords of their email accounts only once in the smartphone, after which their email applications can be accessed at any time. For banking mobile applications, 6 out of 8 participants who access their bank accounts using their smartphones enter their credentials each time they access their bank accounts. Our observations also show that the Wi-Fi networks used by 47% of our participants do not require passwords and are accessible by everyone.

### 4.9 Sensitivity of stored data

Several questions were specifically designed relating to the types of data stored in our participants’ smartphones as well as to their level of sensitivity. Our observations show that 30% of the participants had privacy concerns related to accessing their photos by unauthorized persons. Surprisingly, we also observed that 80% of our participants send their personal photos via WhatsApp without paying particular attention to the risks involved. What’s more, only 27% of the interviewees who back up their data stated that they make backup copies of their photos, whereas 64% pointed out that they only care about taking regular back up copies of their contact lists.

### 4.10 Attitudes toward smartphone loss

We examined the users’ awareness of the consequences of smartphone loss and their perceptions about the risks that they might face if their phones are accessed by unauthorized persons. Only 27% of our participants had experienced smartphone loss incidents, and 25% of them indicated that they use security applications to lock their phones remotely. Furthermore, 37% of the participants believed that no one could access their data if their smartphones were locked. When asked about the damages that they had faced owing to smartphone theft, 37% indicated that they experienced data loss, whereas 63% experienced material damages. Furthermore, when asked about the worst thing that could happen if their personal information were to be accessed by unauthorized individuals, 30% of the participants indicated that they were unaware of the consequences that might occur. However, some of the participants interviewed felt that the worst consequence that might happen is accessing the images stored in their phones by unauthorized persons who might also spread them.

## 5 Users’ behavior: Reasons for adoption

To better understand the users’ risk perceptions, we asked our participants about the reasons that motivated them to adopt or ignore certain behaviors that might affect their security or privacy. Some reported reasons and motives were related to our participants’ lack of awareness and technical knowledge, whereas others resulted from users’ perceptions of threat and costs of adopting safe behaviors. The following subsections include a detailed explanation about the reasons we observed during our interviews.

### 5.1 Reasons for locking/not locking smartphones

Of the 21 smartphone users who lock their smartphones, 57% had privacy concerns related to data access by people they know. However, the reason given by 29% of the users who chose to lock their phones was preventing strangers from accessing their sensitive data. When asked about the motivations behind changing the values of their smartphone locks, 6 participants mentioned that they wanted passwords that are easier to remember, whereas 5 participants indicated that they did not want their children or friends to access their devices. Nine other participants mentioned that they had changed the values of their security locks to match the locks used in other devices or accounts.

In contrast, of the nine participants who decided not to lock their smartphones, six of them believed that locking their devices is a waste of effort because the data contained in their devices are not important. We also noticed that some participants were unaware of the importance of locking their phones and the threats that they might encounter if their data were to be accessed by unauthorized users. Seventeen participants raised some usability issues related to using smartphone locking mechanisms and agreed that there was inconvenience related to unlocking their smartphones whenever they want to access their data or make a phone call. For instance, one of those who did not enable any locking mechanism on her device even though she had privacy concerns related to accessing her data by strangers explained that:

“I do not like to lock my phone. I will start using locking mechanisms if someone take my phone without my permission”

(P22)

### 5.2 Reasons for chatting on WhatsApp

The WhatsApp instant messaging platform was used by 26 participants for chatting and social networking purposes. Most participants indicated that they refuse to chat with unknown persons on WhatsApp for different reasons. Although some had security concerns related to the theft of personal and financial data, other users indicated that they simply do not want to chat with strangers. In contrast, 27% of our participants mentioned that they chat with people who are not listed in their contact lists. They justified their decisions by saying that an unknown person could be one of their friends, colleagues, or family members who has lost his/her phone and was trying to connect via his/her new phone number. Others mentioned that they had situations where their old friends or schoolmates were trying to contact them.

### 5.3 Reasons for sharing photos on WhatsApp

The ease of use and convenience of photo sharing on WhatsApp motivated 80% of the interviewed participants to share their photos without worrying about privacy or security concerns. Some users stated that sharing multiple photos with many users at once and sending live pictures quickly were the main reasons for using WhatsApps photo sharing feature. However, the users who chose not to share their photos on WhatsApp did not mention any particular privacy or security concerns. Instead, they preferred to share their photos via emails because sending their photos via WhatsApp consumes their smartphones’ memories.

### 5.4 Reasons for backing up/not backing up smartphone data

There are four main reasons that motivated 15 participants to regularly back up their data. The main reason reported by 40% of them was experiencing personal data loss incidents in the past. One of those who had decided to make regular backup copies of her data after experiencing the negative consequences that she encountered when she lost her smartphone 4 years ago mentioned that:

“I lost my phone 4 years ago; I lost my important contacts information and that led me to decide to backup my smartphone periodically”

(P11)

Surprisingly, only four participants mentioned that they back up their data because they know that it is important to do so. Another reason mentioned by the participants was related to maintaining their contact lists and smartphone settings. Some also stated that they do not want to start over whenever they start using a new smartphone. In addition, 13% of our participants mentioned that they save backup copies of their sensitive data in another device (such as a PC or a tablet).

On the other hand, half of our participants chose not to back up their smartphone data. Various reasons were observed; however, the majority of our participants stated that they lacked the technical knowledge required to back up their data. Others mentioned that they did not have enough time to back up their smartphone data. Moreover, a few indicated that, even when they purchase a new smartphone, they do not want to fill the new device with the data stored in the old one. One participant said that the data stored in her mobile device were not important for her, and another was unaware of the importance of backing up her data.

### 5.5 Reasons for using smartphone location services

Of the participants who use smartphone location services, 60% stated that they allow social networking applications to access their location information. We observed that most of them were unaware of the risks that they might be exposed to as a result of sharing their locations with others. Their concerns were only related to the consumption of the computing resources of their smartphones (i.e., batteries). Only 20% of the participants did not use these services and were aware that their location information might be misused by strangers. Some said that strangers might know their home addresses and their frequently visited places and leverage these data for some undesired purposes.

## 6 Results and analysis

On the basis of the analysis of the feedback collected during the interviews (the collected data is available in [26]), we show that smartphone users’ avoidance of security and privacy threats is influenced by their threat perceptions, awareness, and knowledge; by their motivation to protect themselves; as well as by the cost of adopting safe behavior, the level of effort required, and the sensitivity of the stored data. Our analysis provided evidence supporting the hypotheses defined in Section 2 and uncovered additional determinants of smartphone users’ self-protective decisions. In this section, we also discuss the links between demographics and security-related behaviors and demonstrate the correlations that emerged and whether they support or oppose the findings of prior work.

### 6.1 H1: Perceived threat

For some security features such as locking and backing up data, we observed that users’ privacy and security concerns were the main drivers behind their decisions to avoid IT threats by adopting protective behaviors. Many participants stated that they made the decision to lock their devices because they knew that they might be exposed to a high degree of risk if someone misused the business or personal data stored in their smartphones. For instance, some users locked their phones because they did not want their personal photos to be seen by strangers in case of smartphone theft or loss. Others mentioned that they had decided to change their security locks on a regular basis or disable their location services when they realized that not doing so made them more vulnerable to privacy and security threats.

On the other hand, we also observed that there was a lack of concern in some situations such as granting permissions to mobile apps and sharing location information in online social networking sites. In many cases, users developed perceptions that the risks associated with their less protective behaviors were not severe, which led them to feel that they were not at risk. In particular, we noted that many users believed that the stored data would not be of any value to adversaries and, therefore, there was no need to take any protective action, such as backing up smartphone data or installing anti-viruses. Other participants also believed that the likelihood of being exposed to privacy or security threats from their trusted friends who knew their security locks was very low, leading them to feel that they would not be threatened by the risks associated with their less protective sharing behaviors.

We also noted that some participants’ misperceptions relating to their susceptibility to privacy invasion resulting from smartphone loss incidents had led them to decide not to take any preventive action. This is because they believed it would be impossible for a stranger to gain access to the data stored in their locked smartphones (e.g., by guessing their security locks). What is more, more than 60% of the participants had decided not to make backup copies of their WhatsApp conversations because they felt that losing access to these conversations would not introduce any threats to them. Comparing this observation to the fact that more than 64% of the participants had confirmed that they make regular backup copies of the contact lists stored in their smartphones, we can clearly see that the participants have perceived the negative consequences of not protecting contact lists to be more harmful than losing access to their WhatsApp conversations, which confirms the direct impact of smartphone users’ risk perceptions on their actual behaviors.

In some cases, we also observed that participants also adopted less protective behavior, although they correctly perceived the threats associated with this behavior. For instance, most of the participants indicated that they do connect to public Wi-Fi networks even though they had security concerns related to their behavior. Furthermore, 80% of the participants stated that they send their personal photos through WhatsApp despite their awareness of their vulnerability to interception by malicious individuals. These observations suggest that the impact of perceived convenience of smartphone features and applications on smartphone users’ actual behaviors might sometimes be higher than that of other predictors of behavior such as the perceived severity and perceived susceptibility of IT threats.

### 6.2 H2: Adoption costs

In most cases, our observations suggested that most smartphone users need cost-effective and usable solutions that maintain their security and privacy. Many participants said that they would not be willing to pay for smartphone protection or backup programs as they perceived that it was not worth paying for protection services and that the built-in protection features of their smartphones were sufficient to protect them against privacy or security threats. In terms of smartphone loss, some users indicated that the monetary value of the lost device is more important to them than the stored data. They had developed the perception that no one could access their data if their phones were locked. They also believed that because the data stored in their smartphones were not sensitive, installing protection programs would be a waste of time and effort. When discussing the importance of locking mechanisms with one of the participants who did lock his device, he explained that:

“The phone is locked and for someone to use it they will have to reset it first, which will delete all existing data. Therefore, the risk is almost zero.”

(P24)

This observation shows that many users who lock their smartphones believe that locking mechanisms are sufficient to protect them against any type of unauthorized access to data stored in their smartphones while not accounting for the negative consequences of other less protective behaviors (accessing their sensitive data while connecting to a public Wi-Fi network, for example).

In other instances, we observed that the effort associated with locking or backing up smartphone devices was one of the main reasons reported by our participants who refused to lock or back up their devices. For smartphone locks, we noticed that some participants preferred pattern locks because they are easier to remember. To minimize the cognitive overheads associated with locking and unlocking smartphones, some users also indicated that they used the same PIN for multiple accounts, whereas others mentioned that they linked their PINs to their bank account numbers or birthdays. For similar reasons, some users preferred not to lock or back up their smartphones, especially if they used more than one device. One of the participants who took the decision to not lock her smartphone anymore after forgetting her PIN and losing access to the data stored in her smartphone justified her decision by saying that:

“I forgot my security lock and my phone was locked as a result of entering wrong PINs three times. At that point, I decided to stop using the security lock.”

(P14)

We also noted that many participants did not back up their smartphones because they perceived it as a time-consuming and effortful activity. For users who indicated that they use more than one smartphone, our findings showed that 60% of them chose not to use any locking mechanism. Furthermore, 80% of them indicated that they do not use backup copies from their previous devices. These findings might suggest that individuals who use more than one mobile device could perceive adopting secure behaviors as time consuming and difficult. Thus, we suggest implementing approaches that simplify the management of passwords, backup copies, and data used across multiple devices.

The perceived convenience of a feature that allows photos to be taken without unlocking smartphones also led many participants to ignore the fact that this feature would allow others to snap pictures without permission. Furthermore, sharing media content via WhatsApp quickly was one of the reasons reported by the participants who chose to use it. Smartphone location services were also preferred by many participants because they make it easier to locate others. All these observations confirm the role of smartphone users’ perceptions of monetary costs, time, and cognitive overheads associated with adopting protective measures on shaping their decisions of whether to protect themselves or not. Therefore, as the perceived costs of taking protective actions increase, smartphone users’ tendency to protect themselves decreases.

### 6.3 H3: Technology awareness

During our interviews, we examined our participants’ awareness of the risks that they might face as a result of adopting less secure behaviors (such as accessing their sensitive data while connected to public networks or sharing their personal photos in WhatsApp). In several cases, we noticed that our participants lacked knowledge of or awareness about the security or privacy consequences of their actions. For instance, only 30% of the users who did connect to public Wi-Fi networks were aware that their actions might be observable by attackers. Additionally, many of the users were surprised when we told them that others who use the same Wi-Fi network might be able to steal any data that they access online. Furthermore, many of the participants who shared their personal photos and location information with others mentioned that they did not know that they might be exposed to privacy or security threats. We also observed that many participants lacked the awareness of the privacy risks that they might be exposed to as a result of granting permissions to smartphone applications.

In addition to the role of threat awareness on shaping the security behaviors of the participants, we also noted that many participants expressed their interest in taking protective actions but mentioned that a lack of knowledge about strategies that could help them protect themselves against potential IT threats prevented them from adopting secure behaviors. Some users indicated that they had no idea about the availability of smartphone security protection programs. Furthermore, most of our participants stated that they have no idea how to stay safe while they are connecting to public Wi-Fi networks. Others stated that they do not know how to back up their devices. One of those who did not make backup copies of the data stored in her smartphone because she lacked the technological awareness that could help her do so mentioned that:

“I don’t know how to make a backup copy, I think it is difficult to make a backup copy for my phone.”

(P14)

These observations support the classification of security awareness of individuals into *“threat awareness”* and *“countermeasure awareness”* provided in [14]. However, our results showed that the impact of *“threat awareness”* on smartphone users’ adoption of protective behaviors was more apparent than the impact of their awareness of countermeasures they could adopt against potential threats.

In other cases, our observations showed that the majority of participants behaved less securely despite being fully aware of the privacy consequences of their actions. For instance, most of the participants who share personal photos via WhatsApp had sufficient knowledge about related data leakage risks. Similarly, at least 87% of our participants who use WhatsApp’s location service feature indicated that they knew the privacy-related consequences of their practices. Interestingly, although most of our participants indicated that they do not trust the content they receive via WhatsApp, they do share their personal data via WhatsApp. These findings showed that adopting less secure behaviors might not necessarily correlate with smartphone users’ lack of awareness. Our results are consistent with the findings presented in [27, 28], which indicated that technological awareness may not directly influence the adoption of secure practices. Therefore, we suggest concentrating on factors that increase the motivation of smartphone users to protect themselves against security threats.

### 6.4 Identified correlations

**Fig 5:**
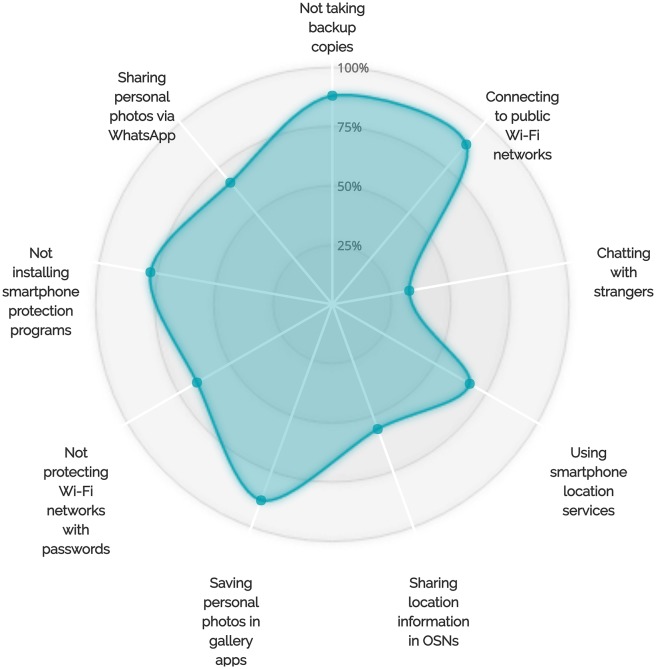
More details on the adoption of other less protective behaviors by participants who did not adopt the locking behavior.

Our findings revealed correlations between locking behaviors, backup behaviors, and sharing behaviors. In particular, our observations suggest that smartphone users’ different unprotective behaviors correlate with each other. For instance, we found that at least 88% of the interviewed users who decided not to lock their devices chose to use public Wi-Fi networks. Another observation showed that 67% of the participants who decided not to lock their devices had no privacy concerns related to sharing their personal photos via WhatsApp. For backup-related behaviors, we observed that at least 88% of our participants who chose not to lock their smartphones never back up their devices (see Fig 5). Interestingly, more than 83% of the users who did not lock and back up their devices had no worries about connecting to public Wi-Fi networks. Furthermore, at least 80% of the users who decided not to lock and back up their smartphones were not interested in smartphone protection programs.

**Fig 6:**
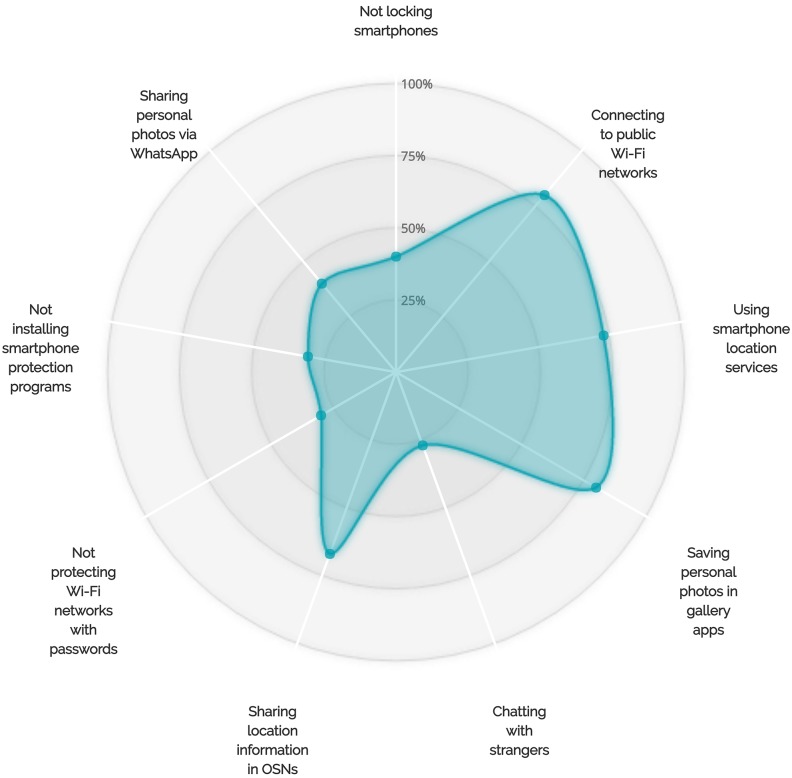
More details on the adoption of other less protective behaviors by participants who did not adopt the backup behavior.

**Fig 7:**
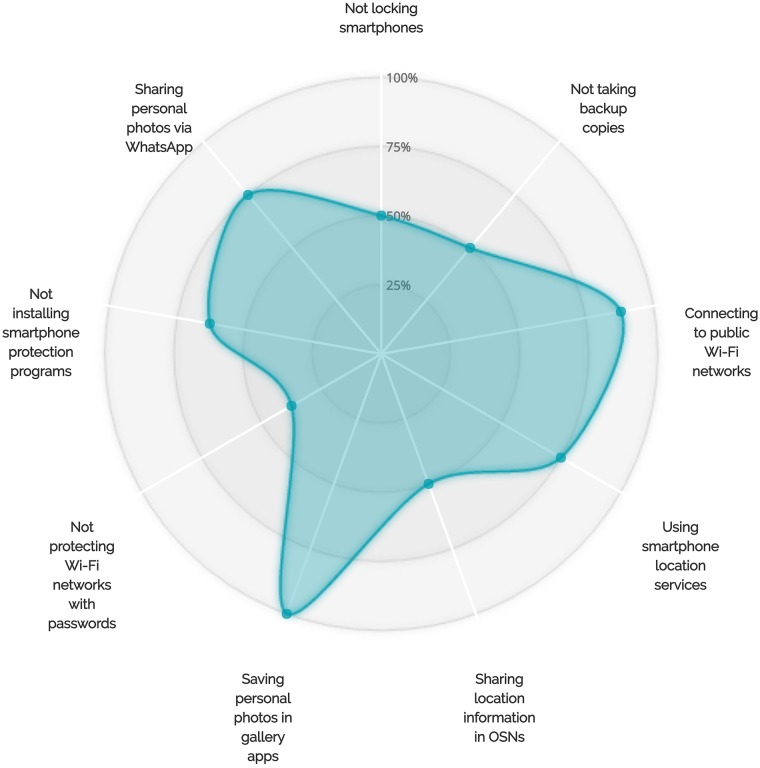
More details on the adoption of other less protective behaviors by participants who did choose to chat with unknown people.

A correlation between locking behaviors and saving personal photos in gallery apps was also observed during our interviews. More than 88% of the users who chose not to lock their phones had saved their personal photos in gallery and camera applications (see Figs 5–7 for more details on the adoption of other unsafe behaviors by those who did not adopt the locking behavior, the backup behavior or did choose to chat with unknown people via WhatsApp). We also observed that backup behaviors correlated with saving personal photos in smartphone gallery and camera applications. More than 12 smartphone users who stated that they do not backup their data admitted that they save their personal photos in gallery applications (see Fig 6).

**Fig 8:**
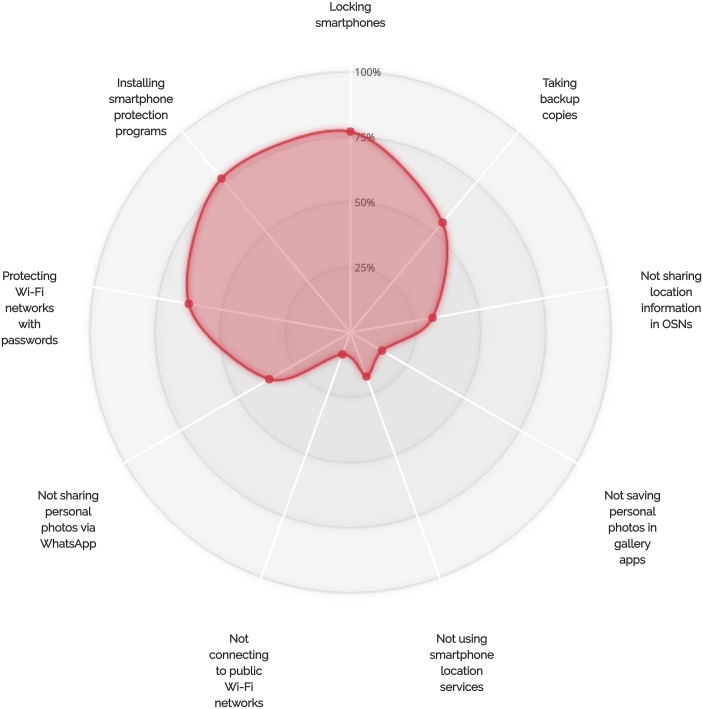
More details on the adoption of other protective behaviors by participants who decided not to chat with strangers.

**Fig 9:**
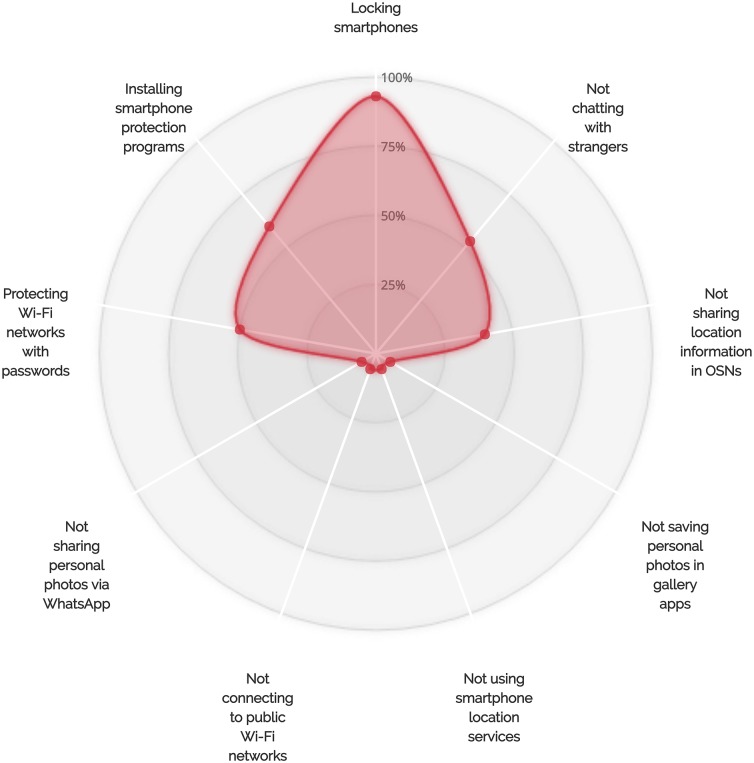
More details on the adoption of other protective behaviors by participants who did adopt the backup behavior.

**Fig 10:**
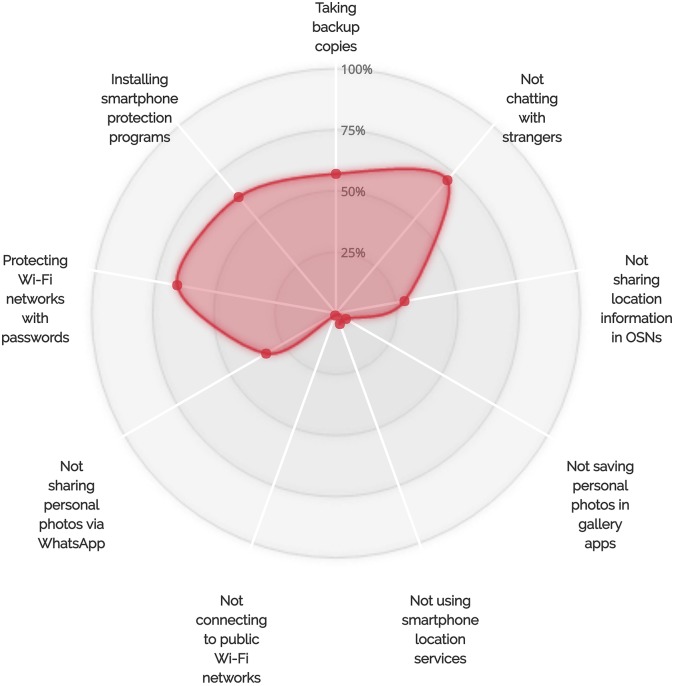
More details on the adoption of other protective behaviors by participants who did adopt the locking behavior.

On the other hand, our results also suggested that there are correlations between some self-protective behaviors of smartphone users. For example, 77% (17 out of 22) of the participants who do not chat with people not listed in their WhatsApp contact lists chose to lock their smartphones (see Fig 8). In addition, at least 82% of the participants who do not talk to individuals not listed in their contact lists and do not share their personal photos via WhatsApp indicated that they lock their smartphones. Furthermore, 93% of the users who chose to regularly back up their devices chose to lock their smartphones (see Fig 9). Sixty-six percent of those who back up their smartphone data and 62% of those who lock their smartphones also indicated that they are interested in smartphone protection programs (see Figs 9 and 10 for more details on the adoption of other self-protective behaviors by those who did adopt the locking behavior and the backup behavior, respectively).

The abovementioned findings clearly suggest that smartphone users’ behaviors correlate with each other. In particular, we conclude that users who chose to protect themselves from certain security or privacy threats may also be inclined to protect themselves from other threats. Similarly, smartphone users who accept the risks from one IT threat might also be inclined to ignore the risks associated with other IT threats by adopting other less protective behaviors. One reason to explain users’ tendency to either adopt or ignore certain protective practices is users’ threat perceptions of corresponding security or privacy consequences. That is, if the perceived severity of certain IT threats as well as the perceived likelihood of experiencing these threats is high, users are expected to adopt protective habits. One observation that supports this idea is the fact that most of the participants who did use their smartphones for business-related purposes had seen the harmful consequences of strangers gaining access to their data, which led them to adopt protective actions. This idea can be supported by findings that showed that more than 67% of the participants who lock their phones indicated that they use their smartphones for performing work-related activities. Furthermore, around 60% of the participants who used their smartphones for work-related activities stated that they make regular backup copies of their data. In contrast, 67% of the participants who decided not to lock their smartphones had installed at most 10 mobile apps from smartphone application repositories without checking whether these apps are secure or not and did not install any mobile banking applications. Furthermore, at least 88% of the participants who indicated that they do not use their phones for work purposes stated that they do not back up their data. These observations therefore suggest that users who do not perform important transactions via their smartphones believed that the perceived severity of their less protective actions and their likelihood of experiencing high degrees of harm are low, leading them to adopt less protective behaviors.

The differences in the percentages of participants who decided to adopt certain protective behaviors and accept the risks associated with other IT threats could be explained by combining the user’s overall perception of threat, adoption costs, and convenience of smartphone features. Many of the participants indicated that they do connect to public Wi-Fi networks, save their personal photos in smartphone gallery apps, and use WhatsApp location services (see Table 2 and Figs 8–10), even though they do adopt a number of self-protective behaviors such as locking smartphones and taking backup copies of stored data. This suggests that the perceived convenience and usefulness of smartphone features might sometimes encourage users to accept the risks associated with using these features, especially if users perceive that they are unlikely to be vulnerable to corresponding security or privacy threats. It is also worth noting that different security or privacy threats have different severity levels, which may lead users to take certain protective measures and decide to leave others. For example, the severity of not locking smartphones or not backing up smartphone data could be higher than the severity of chatting with unknown persons on instant messaging platforms. The perceived likelihood of experiencing security or privacy threats also differs among smartphone users, depending on the type of data they store on their devices and on the level of importance of the transactions they conduct using their devices. The perceived inconvenience or cost of not using insecure smartphone features could also encourage users to accept the risks associated with these features, especially if they offer useful services.

From the percentages of adoption of self-protective security- or privacy-related behaviors shown in Figs 5–10, smartphone users could generally be categorized into self-protective individuals and unprotective individuals based on the level of care they give to their security or privacy. From the observations shown in Figs 8–10, it is clear that some safe behaviors received higher percentages of adoption compared to others (e.g., the locking behavior and the backup behavior). We also note that there are a number of safe behaviors that received low levels of adoption by both groups (i.e., the self-protective group and the unprotective group) such as not connecting to public Wi-Fi networks and turning off smartphone location services. It is also notable that some behaviors received medium adoption levels by both groups (sharing personal photos via WhatsApp, for example). These observations give further support to the fact that the perceived convenience of smartphone features might lead users to accept the risks associated with the use of these features even if they do tend to safeguard themselves against security- or privacy-related threats (e.g., using smartphone location services by the self-protective group). Our observations also show that the perceived costs of adopting certain protection measures (e.g., not connecting to public Wi-Fi networks) might discourage those who belong to the self-protective group from protecting themselves against potential security or privacy risks. The observation relating to having safe behaviors with medium levels of adoption can be explained by the fact that smartphone users differ in the way they perceive the degrees of severity of IT threats and the likelihood of being exposed to these threats. The variations in the levels of self-care smartphone users give to their security or privacy, which are shaped by a combination of factors including the level of technological awareness and the types of transactions performed via smartphones, can also explain why some participants chose to adopt certain safe behaviors while others decided to accept the risks associated with their less protective actions.

### 6.5 Other predictors of self-protective behavior

**Fig 11:**
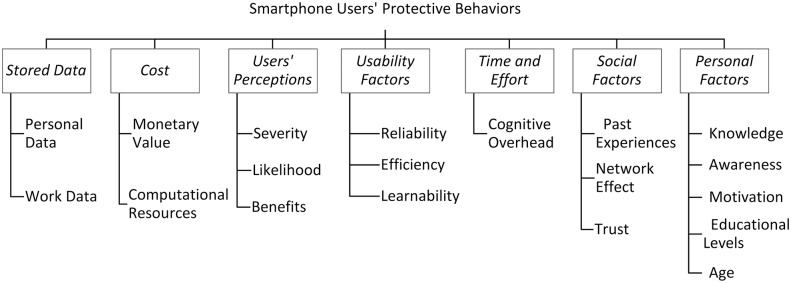
Factors influencing smartphone users’ behaviors.

While qualitatively analyzing the collected data and users’ subjective views about adopting several security behaviors, we were able to identify additional factors that could impact smartphone users’ behavioral decisions. On the basis of our findings and the results presented in prior work, we summarize in Fig 11 the factors that could positively or negatively influence smartphone users’ behaviors.

**Social Factors.** Our results showed that some participants chose to disclose the passwords of their smartphones, emails, or Wi-Fi networks to their friends or family members because they trust them. The majority of our participants also mentioned that they knew the security locks of their brothers’, friends’, or parents’ phones. Others chose to lock their smartphones to prevent their relatives from accessing their data. One participant who did feel threatened by people she knew mentioned that:

“I thought my brother saw my old password, then I just added a dot to my old password”

(P23)

This difference in participants’ decisions on whether to share their security locks with people they trust or not can be explained by the Five-Factor Model (FFM), which is widely applied to explain the differences in personality traits among individuals. According to the *“agreeableness”* dimension of FFM, individuals differ in the level of trust they give to people they know [20, 29]. Consequently, those who score low on agreeableness might feel threatened by people they trust and take protective actions such as creating a new password [20, 29].

We also observed that hearing stories of past security privacy leakage incidents reported by other people motivated some of our participants to adopt secure behaviors. Furthermore, the social network effect played a significant role in encouraging users to share their photos in WhatsApp or share their location information in online social networking sites. When discussing the privacy issues of WhatsApp with some of our participants who shared their sensitive data via WhatsApp, they explained that:

“It’s very dangerous. However, I will still be using WhatsApp application because I can read the news of the whole world!”

(P22)

“As long as WhatsApp is an effective means of communication at both personal and professional levels, it will be difficult to shift to any other app. Being more careful with information communicated via the app will be enough instead of totally abandoning using it.”

(P20)

This observation shows that some groups of smartphone users had decided to share their private data via instant messaging platforms, even though they were well aware of the privacy risks associated with their actions. This could be explained by the fact that those users had decided to accept the risks associated with their less protective sharing habits simply because the services offered by these platforms allowed them to communicate with people they know more easily. This also confirms the fact that the convenience and usefulness of smartphone features and applications might sometimes significantly affect users’ decisions to behave safely.

**Demographics.** Our observations also showed that some demographic groups behaved more safely than others. In terms of locking practice, we observed that the ages of at least 77% of those who chose not to lock their mobile devices were over 32 years. In contrast, 90% of the participants who locked their phones were under 36 years old. Furthermore, the ages of 77% of the participants who decided not to lock their smartphones and did not install any mobile banking application were above 44 years. Another finding indicated that 67% of the participants who chose not to lock and back up their smartphones were older than 43 years.

**Fig 12:**
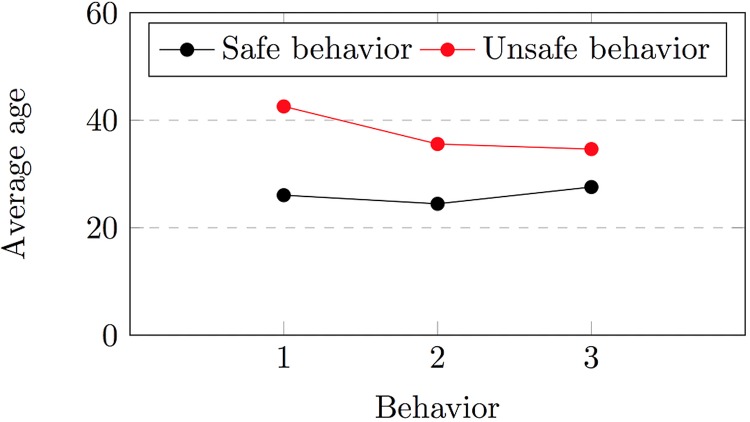
The average ages of participants who decided to adopt, or not adopt, the locking behavior (1), the backup behavior (2) or expressed their interest in installing protection programs (3).

Although our participants’ locking and backup habits correlated with their ages, we did not observe any clear correlation between our participants’ ages and their use of public Wi-Fi networks, smartphone location services, and WhatsApp chatting and photo sharing features. We also observed that the age of 73% of our participants who indicated that they were not interested in smartphone protection programs was over 30 years. The ages of more than 63% of our participants who did not understand how application permission requests correspond to application risks were between 30 and 45 years. Our results confirm the findings of [30], which showed that users’ security behaviors can be grouped according to their ages. However, in contrast with the observations of [30], we noted that younger people tend to have more secure self-protective behavior (see Fig 12).

In other instances, our results suggested that female participants tend to behave more safely when using their smartphones than male participants. For instance, more than 70% of those who expressed their interest in downloading smartphone protection programs, that warn them whenever they install anything harmful from smartphone application repositories, were females. Furthermore, all of those who indicated that they do not use smartphone location services at all were female participants. We also noted that 59% of the participants who refused to chat with strangers via WhatsApp were females. What’s more, the number of female participants who adopted the locking behavior was 33% higher than the number of male participants who adopted the same behavior.

## 7 Platform design recommendations: Toward more secure smartphones

After understanding smartphone users’ behaviors, perceptions, and concerns, we believe that further actions have to be taken by platform designers and application developers to help smartphone users maintain their security and privacy. Smartphone users vary in their demographic groups, technological knowledge, educational levels, and preferences. Because of this, platform designers have to account for these differences and reduce the perceived inconvenience of some security features (e.g., security warnings). As shown in Fig 11, smartphone users’ decisions could be influenced by many factors including the perceived convenience of security features and the time and effort required to adopt secure practices. Therefore, by considering these factors, platform designers can apply multiple platform design modifications to achieve higher security and privacy levels without negatively affecting the perceived convenience of security or privacy features. Some of these modifications can be applied either at the operating system level or at the application level, or by simply installing some plugins. In this section, we propose a set of platform design recommendations to enhance the adoption of security and privacy practices in smartphones.

### 7.1 Security warnings

As emphasized in the literature (e.g., [31–33]), one of the main areas that require a great deal of attention from platform designers is the reduction of the number of times in which the user has to make security- or privacy-related decisions. This can be achieved by reducing the number of times the user has to explicitly lock or unlock her/his smartphone, grant or deny access to the smartphone’s resources, or be presented with security warnings. This, in turn, might increase the level of users’ trust in the usefulness of security features and simplify users’ mental models related to the complexity and sophistication of security features. Therefore, smartphone platforms have to warn the users only when necessary and in situations where real negative consequences might occur.

There are many areas that can be exploited to reduce users’ cognitive overheads and increase the adoption of secure practices. Researchers could develop new techniques that focus on understanding users’ expectations and use users’ contextual information to infer cases in which the users should not be presented with security warnings. For instance, on the basis of the users’ most frequently visited places, an algorithm might infer those Wi-Fi networks that are owned or trusted by the user and implicitly accepts the connection to wireless networks in those places without requesting explicit permissions from the user. New techniques may also emphasize the implicit authentication of users and grant permission to applications that are widely trusted in smartphone application markets and that satisfy well-known security standards.

Previous researchers have suggested that the perceived inconvenience of long warning prompts has led many smartphone users to ignore these prompts [34]. We therefore suggest designing these messages to be concise, understandable, and clear. Warning messages could briefly mention the consequences of the corresponding risky actions. We also encourage platform designers to consider presenting regular feedback to smartphone users at the appropriate time to increase their awareness of the security status of their smartphones. Clearly, inferring some security- and privacy-related decisions might contribute to improving the perceived convenience of smartphone security features.

### 7.2 Security indicators

Platform developers could also add indicators for measuring the security of smartphone applications and for determining whether they are protected from potential intrusions. This might contribute to preserving users’ privacy by analyzing each app’s consumption of users’ data and automatically blocking apps that show abnormal behavior [35]. Furthermore, incorporating security metrics based on user reviews and automated dynamic application security testing into smartphone application repositories is of vital importance since most smartphone users’ installation decisions are affected by app-related information presented in these stores [36]. Inferring security metrics from user reviews would require platform designers to automatically analyze these reviews and ratings and extract information indicating the security levels of the corresponding apps. Evaluating the security levels of mobile applications based on accurate metrics might contribute to reducing users’ cognitive overheads and their frustration related to making security decisions, which, in turn, might positively influence users’ perceptions.

### 7.3 Social dimensions

Consistent with previous findings [28, 37], our observations suggest that there are some social triggers that could affect users’ adoption of protective behaviors. For this reason, we suggest adding social motivations that allow smartphone users to observe the security-related habits of people they know. This might positively impact users’ awareness and knowledge if they are given the chance to discuss security- or privacy-related issues with others. This might also positively influence the behavior of smartphone users who correctly perceived the consequences of their actions but have gotten used to wrong security habits or forgotten how to undertake protective actions. Another possible enhancement is to deliver customized warning messages based on users’ concerns, knowledge, and personal characteristics.

Researchers could also direct their efforts toward understanding users’ preferences, concerns, and perceived security challenges by designing surveys that can be optionally filled by smartphone users once they start using their smartphones to capture each user’s understanding and preferences. The collected feedback is expected to help platform designers build more customized security solutions and adjust smartphones’ settings according to users’ characteristics and preferences. Platform designers could also consider developing mobile device security policies based on the preferences of the target user groups so that users could choose the ones that are suitable for them. In short, smartphone users’ perceptions and behaviors can be shaped and improved by considering the social side of the problem.

### 7.4 Smartphones: Existing security flaws

Platform designers should also focus on identifying and fixing existing security flaws in mobile platforms. If application developers are allowed to upload their apps without serious security scans being performed by a controlled app marketplace (e.g., Google Play [38]), additional security measures should be added to smartphone application repositories. Smartphone platforms should also be modified to force application developers, or at least encourage and educate them, to follow better security practices to avoid the exposure of users’ data. Considerably more attention should be paid to reducing the reliance on the user to make security-related decisions, and the level of isolation between different mobile apps should be increased.

## 8 Related work

Previous research studies investigated how smartphone users’ preconceived perceptions affect the adoption of security mechanisms, addressed users’ attitudes toward locking their smartphones [9, 39], and studied users’ habits related to backing up their devices [9], setting passwords [40], and granting permissions to mobile apps [31, 34]. Prior works also found a strong correlation between users’ adoption of security behaviors and the perceived convenience of the corresponding security features [33, 39]. There are also many researchers studying users’ behaviors in specific security contexts or focusing on addressing the factors influencing the adoption of security practices. The following subsections summarize the literature that addresses users’ security- and privacy-related perceptions and beliefs.

### 8.1 User perceptions: Convenience and usability

The risk perceptions of smartphone users in the medical sectors were examined in [41], and one of the findings showed a strong correlation between the perceived convenience of security controls and practitioners’ intentions of adopting these controls. For perceptions related to smartphone locking mechanisms, previous studies agree that most smartphone users perceive existing authentication schemes as often being inconvenient [9, 39, 42–44]. Harbach et al. studied the unlocking practices of 260 smartphone users and found that users perceived unlocking their devices as unnecessary and time consuming in more than 24% of the cases [39]. Although our results confirm that there is a correlation between the perceived inconvenience of locking mechanisms and their adoption by smartphone users, our study provides further insights by examining the link between locking mechanisms and other smartphone-related security features.

To address the inefficiencies related to smartphone locking mechanisms, some research studies address the usability aspects of locking mechanisms [45, 46], whereas others focus on designing more convenient mechanisms without compromising the security and privacy of the smartphone user [33]. Bhagavatula et al. studied smartphone users’ perceptions related to adopting biometric authentication schemes and found that their participants perceived fingerprint locking mechanisms as more usable than facial recognition mechanisms [46]. Although they found that the adoption rate was higher for fingerprint locking mechanisms, their results showed that both schemes fail to unlock smartphones in some situations (e.g., unlocking with wet fingers). Similarly, researchers investigated how smartphone users perceived the usability and security provided by implicit authentication methods and found that more than 80% of smartphone users were satisfied with the overall performance of these methods [45]. Although the majority of our participants chose PINs as their locking mechanism, our findings showed that there is a need for mechanisms that minimize smartphone users’ cognitive overhead and reduce the time required to lock and unlock smartphones.

As an attempt to improve the efficiency and adoption rate of screen locking mechanisms, Micallef et al. developed an application that employed location sensors to reduce the number of times a user has to explicitly enter PINs or pattern locks [33]. The results of the evaluation presented in [33] showed that users perceived this application as convenient and less annoying compared to existing locking mechanisms. Some researchers investigated the effectiveness of smartphone applications’ permission requests, and whether users’ decisions to install mobile apps are affected by these requests [31, 34]. According to [34], only 17% of the users surveyed took permission requests into consideration before installing their applications. Furthermore, the results presented in [31] showed that 80% of smartphone users perceived some requests as unnecessary. To solve this problem, the authors suggested minimizing the number of permission prompts by deciding whether to grant or deny resource access at runtime and presenting those that the users do not expect [31]. Likewise, Lin et al. implemented an interface that employs crowdsourcing to collect users’ expectations and compares them with the actual security analysis of mobile apps to warn smartphone users only when there was a mismatch [32].

The previous discussion showed that there are many previous research attempts proposing solutions to deal with the inconvenience of security features and increase their adoption. However, all of these studies addressed specific security features independently and without investigating the relationships between different behaviors. Our study attempts to fill this gap by examining the interplay between different security- and privacy-related behaviors. We examined the correlations between several different security practices including locking practices and backup habits, locking behaviors and sharing personal photos in WhatsApp, backup behaviors and storing personal photos in gallery apps and backup behaviors, and connecting to public Wi-Fi networks. By qualitatively analyzing our collected data, we found that several behaviors correlated with each other. We hope our findings will help future researchers in building models that predict the habits of smartphone users and guide platform designers in simplifying the adoption of security practices.

### 8.2 User perceptions: Misconceptions about security

Prior research studies have also addressed the relationships between users’ perceptions, belief, and behavior [47–49]. By investigating users’ perceptions and security practices in a developing country, Chen et al. found that Ghanaian users believe that password systems can protect them against security threats [47]. Muslukhov et al. found that smartphone users had privacy concerns related to backing up their sensitive data in cloud-based storage systems and preferred to store their backups locally (e.g., using hard disks or memory cards) [48]. This misconception about cloud-based storage systems was also observed in our study, which was conducted in a totally different cultural context. This might suggest that different cultural groups might have similar security perceptions and behaviors. Another study found that people perceived smartphones as less secure than personal computers, and these perceptions affected their behavior by choosing not to use their mobile devices for accessing sensitive data [36].

### 8.3 User perceptions: Influencing factors

Other researchers focused on examining whether users’ protective behaviors are influenced by their technical knowledge and awareness of security threats [30, 50]. Wash et al. found that users’ security perceptions can be grouped according to their demographic groups and technological knowledge [30]. For instance, their observations showed that people with lower educational levels tend to make simpler security decisions [30]. However, the findings presented in [27] showed that users’ understanding and technical knowledge of how the Internet works do not directly impact their adoption of security behaviors. Our observations are partially consistent with the results of [27, 30] in that users’ behaviors can be grouped according to their age groups. However, our results also showed that knowledge and awareness partially impact the adoption of correct security practices. On the basis of theoretical approaches, Blythe et al. studied the factors affecting employees’ security-related behaviors in the workplace [51]. Consistent with our findings, Blythe et al. found that employees’ behavior was affected by their knowledge, previous experience, and the perceived time and effort related to adopting specific security practices [51].

Other authors argue that users’ protective practices are greatly affected by their motivation to protect themselves against information security threats [52]. This idea was also supported by the findings of [28], who examined the social factors that affect users’ perceptions and behavior about security and privacy. The majority of the users interviewed in [28] were motivated to adopt self-protective behavior as a result of observing the security-related practices of their friends. The authors suggested implementing approaches that focus on raising individuals’ motivation to behave securely and acquire the knowledge required to protect themselves [28].

### 8.4 Security and privacy risks

Other researchers have directed their efforts toward investigating the privacy- and security-related implications of smartphone users’ risky practices. Some studies focus on determining the types of sensitive data that could be extracted by malicious attackers from their victims’ mobile devices, whereas others have proved the feasibility of intercepting mobile communications and extracting personally identifiable information from the exchanged traffic. In [53–55], researchers demonstrated the vulnerability of popular and widely used mobile VoIP applications to unauthorized interception. In particular, they found that users’ communications over VoIP applications are not always protected, making it easy for individuals with malicious intentions to decode intercepted communication. What makes the matter even worse is that some VoIP applications do not provide documentation that could make users aware of the fact that their video, audio, or text communications over some VoIP platforms are not encrypted [53]. It has also been found that most classes of personal data collected by mobile applications can be recovered from the traces left in users’ smartphones [53, 56–59]. In [57], for instance, researchers were able to recover important details related to bank accounts and financial transactions from mobile banking applications. It was also found that remnants of social networking applications installed on smartphones (such as Twitter and LinkedIn applications) could be utilized for revealing personal information about smartphone users and understanding their social contexts [60]. Cloud storage mobile applications have also been found to store important information that could be useful for inferring knowledge about the types of files users share and for constructing useful information about users’ activities [61–67]. In cases of smartphone theft or loss, combining different types of data left by or exchanged through different smartphone applications could help attackers build profiles of their victims and gain unauthorized access to sensitive data. Attackers could also use their victims’ compromised smartphones to gain unauthorized access to valuable data stored in other devices such as paired personal computers or wearable devices [68, 69].

Given the direct role of human factors in driving individuals to take correct preventive and protective measures, the rise in the amount of personal data stored in smartphones and the fact that many smartphone applications gain access to resources that are not necessarily needed for them to function [70, 71], we expect the results of our analyses of users’ behaviors and risk perceptions to help platform designers to design secure and privacy-preserving solutions.

## 9 Limitations and future work

Despite the fact that this study has focused on analyzing security and privacy perceptions and behaviors of only 30 Android users, we believe that the results presented in this work offer an improved understanding of the factors that cause smartphone users to accept the risks of privacy and security threats or decide to undertake protective actions. We also encourage future researchers to replicate our study on a larger sample that includes users of other smartphone operating systems. Further, as 86% of our participants belong to one country, we believe that that our research is a valuable source for understanding whether there are any cultural properties that can explain users’ decisions to protect themselves as the results presented in this paper provide the foundations that could facilitate studying the behavioral differences between smartphone users belonging to different countries.

Future directions also include aligning smartphone security features with users’ expectations such as developing new mechanisms that preserve users’ security while maintaining an efficient consumption of smartphone computing resources. Future studies could investigate whether smartphone users’ confidence in their abilities to protect themselves can relate to their self-protective actions. We also leave to future researchers the task of studying whether smartphone users’ perceptions of the effectiveness of existing security or privacy-preserving solutions can affect their decisions to engage in protective behaviors. We also argue that smartphone platforms sometimes leave no option for the user but to adopt unsafe practices. We therefore encourage platform designers to take advantage of the recommendations we provide in Section 7 to improve support for user-oriented privacy and security features. As well as considering our suggestions for improving the security of smartphones, we believe that smartphone users are the weakest line of defense against many IT threats, and we therefore emphasize the importance of conducting further research for uncovering additional predictors of security and privacy behaviors.

## 10 Summary and concluding remarks

Smartphones are susceptible to many attacks because they store data of varying sensitivity, connect to public and private networks, and deal with multiple types of sensors. There were a number of studies that focused on researching users’ privacy and security concerns. Previous research also addressed users’ perceptions related to locking mechanisms, backups, and application permission requests. To the best of our knowledge, the literature lacks studies that examine the relationships between different security behaviors and, at the same time, address users’ perceptions related to connecting to public Wi-Fi networks, using mobile instant messaging platforms, saving personal photos in gallery applications and taking advantage of smartphone location services.

Our paper focuses on studying the relationships between different smartphone security practices by presenting and discussing the results of 30 qualitative interviews. By comprehensively examining users’ behaviors related to utilizing many smartphone security features, we confirm, extend, and refine the findings of prior work by providing evidence that users’ protective behavior is influenced by many factors, including users’ awareness and motivation, their personal characteristics, and their perceptions. We also identify the factors that could influence users’ adoption of security-related behaviors. These include social triggers, the time and effort required to adopt certain practices, and the importance of stored data. Our observations also showed that some behaviors correlate with each other (e.g., locking behaviors and backup habits) and that younger people tend to behave more securely while using their smartphones. We believe that the recommendations we proposed for platform designers and application developers hold a potential to improve the security of smartphones by focusing on the factors that might drive significant behavioral changes.
